# A model to rate strategies for managing disease due to COVID-19 infection

**DOI:** 10.1038/s41598-020-79817-7

**Published:** 2020-12-31

**Authors:** Shiyan Wang, Doraiswami Ramkrishna

**Affiliations:** Davidson School of Chemical Engineering, Purdue University, West Lafayette, IN 47907 USA

**Keywords:** SARS-CoV-2, Viral transmission

## Abstract

Considering looming fatality and economic recession, effective policy making based on ongoing COVID-19 pandemic is an urgent and standing issue. Numerous issues for controlling infection have arisen from public discussion led by medical professionals. Yet understanding of these factors has been necessarily qualitative and control measures to correct unfavorable trends specific to an infection area have been lacking. The logical implement for control is a large scale stochastic model with countless parameters lacking robustness and requiring enormous data. This paper presents a remedy for this vexing problem by proposing an alternative approach. Machine learning has come to play a widely circulated role in the study of complex data in recent times. We demonstrate that when machine learning is employed together with the mechanistic framework of a mathematical model, there can be a considerably enhanced understanding of complex systems. A mathematical model describing the viral infection dynamics reveals two transmissibility parameters influenced by the management strategies in the area for the control of the current pandemic. Both parameters readily yield the peak infection rate and means for flattening the curve, which is correlated to different management strategies by employing machine learning, enabling comparison of different strategies and suggesting timely alterations. Treatment of population data with the model shows that restricted non-essential business closure, school closing and strictures on mass gathering influence the spread of infection. While a rational strategy for initiation of an economic reboot would call for a wider perspective of the local economics, the model can speculate on its timing based on the status of the infection as reflected by its potential for an unacceptably renewed viral onslaught.

## Introduction

The pandemic of coronavirus (SARS-COV-2) infection has gripped the world with unparalleled anxiety. An alarming number of deaths have occurred within the short span of a little over 4 months! In US, more than one hundred thousand have died at the time of compiling this article with prospects of many more in the horizon. Despite the epidemic slowing, it appears to be abating at an unacceptable rate. There has been a scramble for controlling the spread of infection by people of various backgrounds including medical professionals, scientists, engineers, economists, the media, and political leaders. Although considerable insight has accumulated over efficient ways to confront this cataclysm^1,2^, much more remains to be learned about the disease transmission, its treatment, and prevention by a suitable vaccine for the future. While the government has taken actions to relieve the economic burden of coronavirus on certain industries, businesses, and American workers (e.g., paycheck protection program), the looming prospects of an economic breakdown of catastrophic proportions are a further complication that must also somehow influence the mode of confrontation of the pandemic.

An essential prerequisite to facing the coronavirus pandemic is understanding of the various factors that have a potential contribution to limiting the spread. The spread of infection occurs in multifarious ways. Thus one that is cited the most frequently is spread of the virus through droplets from coughing and sneezing^3^. Another is from unwitting contact with infected surfaces^4^ such as glassware, boxes and so on. Intimate contact through handshakes and hugs are even more efficient ways to transmit infection. Each occurs through different scenarios that must be envisaged with their respective frequencies of occurrence for a model formulation. For symptomatic disease associated with a pathogen transmissibility (marked by a basic reproduction number), different transmission routes are aligned to their implications for prevention; specifically, there may be four categories: symptomatic transmission, pre-symptomatic transmission, asymptomatic transmission, and environmental transmission. Given recent evidence of SARS-CoV-2 transmission by mildly symptomatic and asymptomatic persons^5^, its incubation period is about 5.1 days and about 12 days of infection from exposure to symptom development (latent period). Therefore, unusually long term of latency period and pre-symptomatic transmission could have important implications for transmission dynamics^6^.

Analysis of data accumulated from numerous sources have provided the general features of the spread in terms of when to expect the peak infection rate and what it takes to flatten this curve. Yet this understanding must be said to be qualitative without notable predictive features. A mathematical model is presented here of the spread of coronavirus (COVID-19) in terms of three parameters that control the rate of its spreading and flattening the infection rate curve when intervention by a vaccine is not available. Our model is concerned with a specific geographic domain of the United States with a given population of specified density (number per unit area) of which a fraction is initially infected. The infected population contributes virus within the domain which, for the present, is completely isolated from other domains. The spread of infection within the domain depends on the uninfected population and occurs at a rate governed by the extent of protective measures adopted to avoid infection from those infected. This spread obviously depends also on the viral population in the domain which grows by contribution from the infected (exhaled droplets, aerosol, contaminated surfaces, and possibly fecal-oral contamination^7^) and disappears by death/isolation etc. We should note that while there are numerous reports on reinfection of COVID-19^8^, majority of recovered patients retain certain immunity against the virus.

**Table 1:** Emerging indicators from pathogen system.

Dimensionless variables	Definitions
$\alpha =\frac{k_vN_o}{kV_o^2}$	Fractional rate of viral growth during the (average) infection time with ${{V}_{o}}$ viral population; $k_v$is the rate constant for production of virus from the infected; *k* is the rate constant for transfer of infection
$\beta =\frac{k_v'}{kV_o}$	Viral death rate during (average infection time); $k_v'$ is the rate of death of virus
$\gamma =\frac{k_r}{kV_o}$	Removed rate of infected patients relative to infection rate with ${{V}_{o}}$ viral population; $k_r$ is the rate of removed population (either by death or recovery)

**Figure 1:**
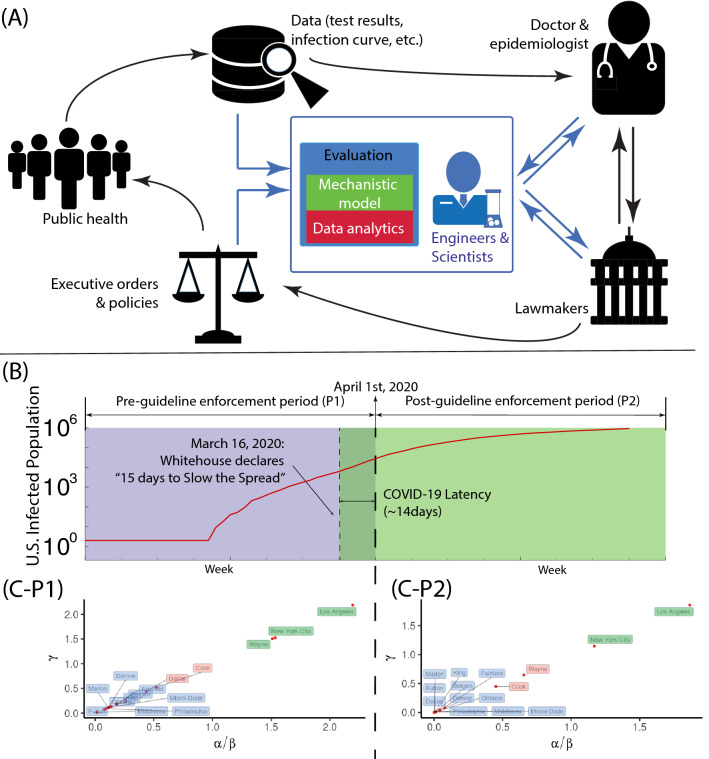
Parametric study on coronavirus infection in United States. (**A**) By incorporating both mechanistic modeling and data analysis (in blue) into the traditional workflow of the policy making (in black), a refreshed framework forms a three-way communication among expert (doctor/epidemiologist), engineers/scientists and lawmakers, thus improving the implementation of health policies against the infectious disease. (**B**) The timeline of total population with COVID-19 positive in conjunction with the policy of “15 Days to Slow the Spread” in the United States, in which the infection period is divided into the pre-guideline enforcement period (P1) and the post-guideline enforcement period (P2). (**C**) The phase space in terms of $\alpha /\beta$ and $\gamma$ is plotted for the leading infected counties in first fifteen states: P1 duration and P2 duration. The phase space is segregated as three regions: ‘severe’ (labeled as green; $\alpha /\beta \in (1.0,\infty )$), ‘moderate’ (labeled as red; $\alpha /\beta \in [0.4,1.0]$), and ‘mild’ (labeled as blue; $\alpha /\beta \in (0,0.4)$).

Our goal here is to find a suitably simple framework to produce a mathematical model that contains a limited number of parameters which can be readily identified from gross observations. Furthermore, they should relate in some way to various strategies that may be envisaged to control the spread of infection. To simulate both dynamics of viral and infected population, the modeling of the system in a considered geometric domain can be abstracted as its dimensionless form

1 $$\begin{aligned} \frac{dx}{d\tau }= (1-x)y-\gamma x, \end{aligned}$$

2 $$\begin{aligned} \frac{dy}{d\tau }= \alpha x-\beta y, \end{aligned}$$

where $x=n/N_o$ is the infected population density (*n*) normalized by the population density in the domain ($N_o$), $y=V/V_o$ is the dimensionless viral population density, $\tau =t/T_{\text {inf}}$ is the time scaled by the average time ($T_{\text {inf}}$) for an individual to be infected; The explanations of both dynamic equations are elaborated in the methods section. Three dimensionless parameters (see physical interpretation of $\alpha$, $\beta$, $\gamma$ in Table 1) presented in the above differential equations compare the rates of different processes and have the capacity to control the spread of infection. Daily infection data must be fitted to the model by appropriate choice for the values of the dimensionless parameters (see Fig. S1 in supplementary material). The socio-economic behavior has diversified the dynamics of the infection curve; Furthermore, major regulatory governmental strictures may enforce more discipline in public behavior thus seriously affecting the parameters. This effect, it must be conceded, is buried in subtle empiricism of the model that we must seek to unearth. In doing so, we emulate the currently popular practice of machine learning towards estimating the parameters in each domain to assess the local government policy. We note that the current study focuses on the measurement of policy effectiveness during the pandemic; the policy measures during the early stage of disease transmission have been document by the World Health Organization^9,10^. In this regard, the informative results delivered by combining both approaches (i.e. mechanistic model and machine learning) could promote effective policy implementations against the transmission of disease (Fig. 1A). In Fig. 1B, the national scale social distancing is undertaken with the administration guideline “15 Days to Slow the Spread” that divides the pre-guideline enforcement period (P1) and the post-guideline enforcement period (P2). Furthermore, to consider the heterogeneity of the population density, we model the infection dynamics in the leading county of every state (50 states plus Washington D.C.) Within different periods and regions, their parameter values will reflect the quality of management of the spread of infection in the area under consideration. The different mechanisms of transmission of infection may operate to varying extents in different areas depending on how the infection is managed locally. Thus one must regard the model as only “broadly” mechanistic and the relationship of model parameters to different strategies would be somewhat diffuse. Therefore, in connecting the model to guide strategies we resort to a statistical methodology based on machine learning tools, which could overcome the limitation just mentioned.

## Results and discussion

### Role of parameters in spread of infection

We first examine role of model parameters in the spread of infection. The P1 duration reveals the period of pathogen transmission with limited prevention in the United States. The early state of virus transmissibility can be characterized by ‘R-naught’ (R0), which is the basic reproduction number. Our estimate of R0 is about 2.8 (the median from data is 2.75; our model is 2.90; see the calculation method in supplementary material) whose transmission is stronger than influenza (R0: 1.4–1.6)^11^ and weaker than Measles (R0: 12–18)^12^. The speed of infection of an individual would depend on the value of $T_{\text {inf}}$: a large $T_{\text {inf}}$ would imply a longer real time and thus a slower rise in infection. For instance, in New York at P1 period without government policy intervention, it typically takes about $T_{\text {inf}}\sim 10$ minutes to infect an individual. With the implementation of government policy about social distancing, in P2 period, $T_{\text {inf}}$ increases 25 times, and the approximation of an individual infection takes about 4 hours! To measure parameters ($\alpha$, $\beta$, $\gamma$), it is purposeful to examine the steady state solutions of the model represented by Eqs. (1) and (2). We show that the only relevant solution is $\tilde{ x}=1-\gamma (\alpha /\beta )^{-1}$ and $\tilde{ y}=\alpha /\beta -\gamma$ where the projected total infected population ($\tilde{ x}$) and viral population ($\tilde{ y}$) are determined by $\alpha /\beta$ and $\gamma$. Parameter $\alpha /\beta$ represents the ratio of viral growth rate to its death rate, which represents the extent of environmental virulence. Fig. 1C shows that severe virulence environment (large $\alpha /\beta$) are associated with the large counties (i.e. Los Angeles-CA (P1,P2), New York City-NY(P1,P2)). In particular, Wayne county at Michigan State shows significant improvement (severe$\xrightarrow {}$moderate) as the proper social distancing is taken and thereby there would be a significant reduction of the virus in circulation. In general, counties with populous majority remain as small virulence during the entire period (Fig. 1C). Parameter $\gamma$ represents the removal rate of infected patients (by recovery/death). Our model implies that $\gamma$ is associated with $\alpha /\beta$ positively: despite the infection, its percentage in each county remains low (e.g. the percentage of infection at New York City is about $O(10^{-2})$); Therefore, $\gamma (\alpha /\beta )^{-1}\sim 1$ and always $\gamma <\alpha /\beta$. Here, we ought to demonstrate two significant points: (1) mathematically, $\gamma <\alpha /\beta$ is the necessary and sufficient condition for the stability of the solution; (2) as the difference between $\gamma$ and $\alpha /\beta$ becomes smaller, the eventual infected population is smaller. The most direct way of containing infection depends on the availability of effective vaccines and therapies that can raise the value of $\gamma$. However, we should note that, without further modification, the current model would not directly account for the possibility of using experimental prescriptions such as Remdesivir recently authorized by Food and Drug Administration (FDA)^13^.

**Figure 2:**
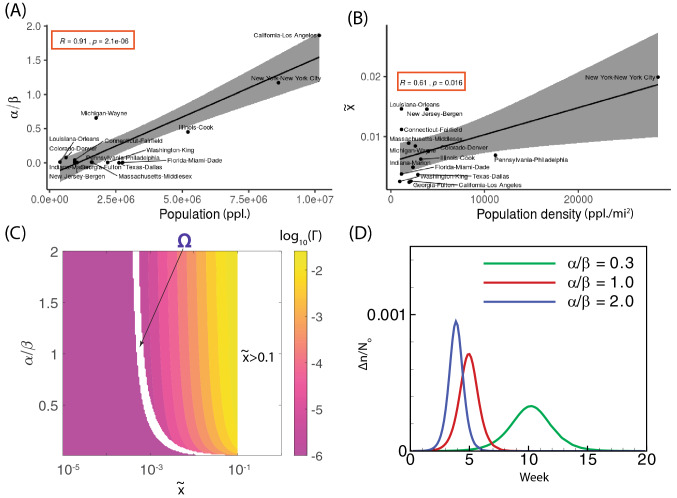
Relationship between county population and ${\alpha /\beta }$ & ${{\tilde{x}}}$. (**A**,**B**) The statistical correlations (R: Pearson correlation coefficient) are displayed for (**A**) virulence ($\alpha /\beta$) vs. the corresponding county population and (**B**) the projected infection fraction (${\tilde{x}}$) vs. the corresponding county population density (sample number is 15). The solid line is the linear regression with a grey shaded area of 95% confidence interval. (**C**) A phase diagram in terms of $\alpha /\beta$ and ${\tilde{x}}$ for the peak infection rate $\Gamma$; domain ‘$\Omega$’ represents the region in which the peak infection is not reached and Region $\{{\tilde{x}}>0.1\}$ indicates the unlikely space where total infection exceeds 10% of the total population in the domain considered. (**D**) The effect of dimensionless parameter $\alpha /\beta$ on peak infection is studied with ${\tilde{x}}=0.01$. For (**C**,**D**), the simulations are conducted for a duration of 1 year (365 days) with $T_{\text {inf}}=40$ min.

### Indicators of COVID-19 transmissibility: $\varvec{\alpha /\beta }$ & $\varvec{{\tilde{x}}}$

Based on our model, we propose two indicators $\alpha /\beta$ and ${\tilde{x}}$ to characterize the infection dynamics. Reducing $\alpha /\beta$ is accomplished by slowing the viral transfer from the infected to the uninfected which can be accomplished by several ways such as social distancing (individual precautions can be washing, wearing masks, physical distancing 6 or 12 feet, and so on). In Fig. 2A, we find that $\alpha /\beta$ is strongly correlated with the county population (R = 0.91, p = 2.1e−6; p<0.05 considered as significant), which is consistent with the physical explanation of $(\alpha /\beta )\propto (N_o/V_o)$ in Table 1; given any domain, $N_o/V_o$ increases with large population number but independent of population density $N_o$. On the other hand, the projected total infection fraction ${\tilde{x}}$, which characterizes the pathogen transmissibility, is positively correlated with the county population density (R = 0.61, p = 0.016 in Fig. 2B) because of $N_o$; the transmission of the infection becomes faster when the population density is high. Other factors such as weather (temperature, humidity) remain insignificant (weak correlation) to the infection dynamics (see Fig. S2 of the supplementary material and reference^14^; also see the effect of temperature on the of suspected, confirmed and death of COVID-19^15^). Figure 2C exhibits how parameters $\alpha /\beta$ and ${\tilde{x}}$ affect the peak infection rate. The peak infection rate represents the stage beyond which the infection rate will drop. It is now possible to address the much debated strategy of “flattening the curve” by lowering peak infection rate. To reduce the transmissibility (i.e. lower ${\tilde{x}}$), the peak infection rate has to be small (see Fig. 2C). Our model recommends that this is accomplished when $\alpha /\beta$ is low, suggesting the reduction of virus circulation (Fig. 2D).

**Figure 3:**
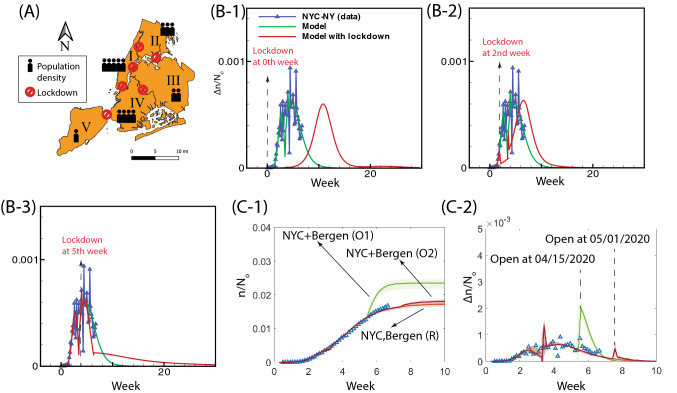
Effect of lockdown and reopening economy on the spread of infection in New York City. (**A**) The strict policy of lockdown is assumed at the borders of five boroughs—Manhattan(I), the Bronx (II), Queens (III), Brooklyn (IV) and Staten Island (V) within New York City. (**B**) The date of initiating lockdown affects daily positive cases $\Delta n$ in New York City scaled by total population density $N_o$, where the start of policy enforcement is set at different initiation points: zeroth week (B-1), 2nd week (B-2), and 5th week (B-2), respectively. (**C**) Modeling of infected population (C-1) and increment (C-2) by considering different opening periods for New York City and Bergen county (inner New Jersey circle): ‘NYC+Bergen (O1)’ indicates the reopening economy at 5.5th week; ‘NYC+Bergen (O2)’ indicates the reopening economy at 7.5th week; ‘NYC, Bergen (R)’ indicates the economy remains closed. In (**C**), $N_o$ represents the averaged resident population density of both New York City and Bergen (New Jersey); Blue symbols ‘$\Delta$’ are the data of the infected population within New York City and Bergen (New Jersey); Plots with shaded area are the modeling results where solid lines represent the median of the prediction and the shaded area indicates the uncertainty. The zeroth week is set at the moment when the total number of infections at the New York City is ten.

### Impact of lockdown: New York city

We now proceed to model the effect of lockdown on COVID-19 transmissibility in New York City, as an example. Additionally, considering recently published policy of “Opening Up America Again” by the white house administration, we will study the effect of reopening economy on the dynamics of transmissibility in the New York metropolitan area in next section. Here, we consider the influence of lockdown policy at New York City, where the isolation is determined by the geographic constraint of five boroughs (the Bronx, Brooklyn, Manhattan, Queens, and Staten Island) within the New York City (Fig. 3A). From Fig. 3B, our model suggests that the mitigation brought about by lockdown is sensitive to the moment of implementation; an early enforcement of lockdown could delay its occurrence. Our study also shows that if the implementation happens after the peak infection, the strategy of slowing works less efficiently. However, the lockdown likely results in a long-term dynamics; it not only impacts the economic damage, but also brings the negative mental health to children and college students due to depression, and anxiety^16^, which calls for interventions and preventive strategies^17^.

**Figure 4:**
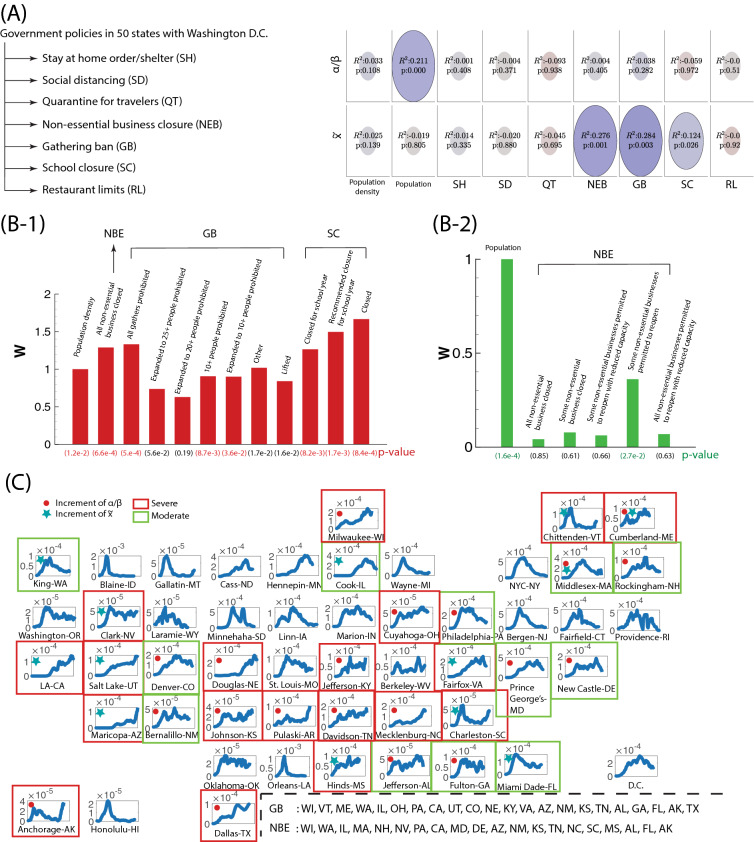
Evaluation of policies on COVID-19 transmissibility. (**A**) The relevance and significance of individual policy and population dynamics (population and population density) on model parameters $\alpha /\beta$ and ${\tilde{x}}$, characterized by adjusted R-square and p-values, respectively. In the diagram, the color of the ellipse represents the value of adjusted $R^2$ while the size of ellipse accounts for p-value. (**B**) The regression analysis of government policies and local population dynamics against (B-1) the projected eventual infection fraction ${\tilde{x}}$ and (B-2) virulence $\alpha /\beta$. The extent of influence from individual factor is reflected by the corresponding weight (*W*). In (**B**), the weights are normalized by the first factor. (**C**) Upon economy opening, the modification of polices impacts the trend of infection curve. We can identify the states with the surge of positive cases by the prediction of $\alpha /\beta$ and ${\tilde{x}}$ using machine learning: the blue plots represent the daily increment $\Delta n$ (7-day running averaged) normalized by the total population density $N_o$ in the leading county of all fifty states (including D.C.) For all emerging counties in each state, a red filled circle is recognized by the elevation of $\alpha /\beta$ from the prediction of the machine learning; A green filled star represents the increase of ${\tilde{x}}$; the red square implies the severe situation based on the plateau/upward-trending daily growth and the yellow square indicates the moderate scenario based on the downward-trending daily growth curve.

### Reopening economy on second wave: New York metro area

During this pandemic, the financial center of the world–New York City area has been hit by massive layoffs and anticipates looming recession^18^. This situation, spells some urgency for reopening the economy and resuming normal daily activity. However, we stress that opening the economy has to be cautious to the possible appearance of a second wave thus making the timing of the reopening very important. To simulate the impact of normal daily activity on the current dynamics of infection, we study the transmissibility in both New York City and Bergen County (Inner New Jersey) within the metropolitan area. These two regions represent the most active interactions in the United States (leading out-computing in the metro area, NYC Planning 2018) and yet both have the leading coronavirus infections in their states. In the model, we relax the current government restraints and resume normal daily operations and activities, which allows the model to consider the worst scenario of the infection curve. Figure 3C shows that the economy reopening (with the least precaution) inevitably brings the second wave and thereof more mortality. However, the extent of infection outbreak can be drastically reduced by delaying the opening date (35% increase at 5.5th week vs. 4% increase at 7.5th week). We note that an effective policy intervention may reduce the drastic increment of the infected population. In the next section, we discuss how to quantify the effectiveness of current implemented policy on coronavirus transmissibility.

### Influence of policy measures in states

With the U.S. administration declaring the social distancing guideline since March 16th, local governments have implemented more than 300 executive orders in fifty states, Puerto Rico, the District of Columbia, Guam, and the Virgin Islands. The executive actions and policies are related to declarations of states of emergency, school/business closure, prohibition of mass gathering, stay at home order, etc. Central issues stand as the effectiveness of ongoing individual policy is unclear. In our study, while the coronavirus transmissibility is strongly influenced by the local population dynamics (Figs. 2A,B), the statistical inference suggests the relevance of several ongoing policies on the coronavirus transmission (Fig. 4A). With our model empowered by machine learning tools, we perform the regression of both parameters $\alpha /\beta$ and ${\tilde{x}}$ based on all policy influences, where the detailed description of data sample for policy is shown in Table S3-1 of the supplementary material. By examining the weight associated with each policy measure and its significance (p-value) in Fig. 4B-1, we should conclude that factors such as non-essential business closure, gathering ban and school closure possess strong impact on ${\tilde{x}}$ (adjusted-R2 = 0.59, p = 2e−6; also see Fig. S3 in the supplementary material), which represents the total infected population. Both gathering ban and school closure emphasize the activity of population in young age, which is consistent with the recent finding that young people play a vital role in spread of COVID-19^19,20^. For virulence environment ($\alpha /\beta$), while the severity of coronavirus spread is largely determined by the local population number, nonessential business closure plays a role in its attenuation effort among other considered policies (adjusted-R2 = 0.30, p = 1e−3; see Fig. 4B-2 and Fig. S4). With the context of reopening the economy (since May 1st 2020), there are several reports on the surge of coronavirus infection in multiple states and most contagious region remains at the leading counties. Policies on both certain non-essential business limitation and gathering ban have been revised in almost all states (note: schools remain closed across the United States; see Table S3-2 in the supplementary material). Our model enables to incorporate these policy changes to predict and diagnose many states having surges of positive cases (Fig. 4C). By predicting the increase of $\alpha /\beta$ and ${\tilde{x}}$ using machine learning, we find that the strong surges (marked as ‘severe’ in Fig. 4C) in states like Utah, Nebraska, Ohio, Kentucky, Texas and Virginia could stem from the relaxation of the gathering ban; Nevada, North Carolina, South Carolina and Mississippi have observed high daily positive cases, which is interpreted by our model as due to the non-essential businesses; We also find that several states (e.g. California, Wisconsin, Arizona, Alaska, Tennessee, Maine, etc.) have strong infection due to both factors (non-essential business closure and gathering ban). Our model captures all currently emerging states, indicating significant impact of government policy on the spread of coronavirus when a vaccine is unavailable. In Fig. 4C, several states (marked as ‘moderate’) should be cautiously optimistic when relaxing social distancing measures despite downward trending of daily positive number because we observe increases in either $\alpha /\beta$ (elevated virulence) or ${\tilde{x}}$ (increased projected total infection number) in our model. In particular, the state of Massachusetts should retain strong measures (increments in both $\alpha /\beta$ and ${\tilde{x}}$).

## Conclusions

In this report, we have proposed a new mechanistic model describing the transmission of COVID-19 in the United States. Our model is established in conjunction with administration policy, from which we propose two significant parameters. The parameter $\alpha /\beta$ quantifies the severity of the coronavirus circulation, and the parameter ${\tilde{x}}$ represents the projected total infected fraction. To be consistent with CDC county-by-county guideline, we studied the infection dynamics of the leading county in each state. By examining the peak infection rate, our suggested strategy of ‘flattening the curve’ has to deal with lowering $\alpha /\beta$, towards drastically diminishing the virus population in the environment. Our study of lockdown suggests that it be implemented before the peak infection rate, since its arrival can be sensed well by the parameters. We have quantified the impact of current social distancing policies with $\alpha /\beta$ and ${\tilde{x}}$, suggesting that polices such as, restrictive non-essential business closure, a ban on gathering, and that of school closure are critical. This may strongly associate with the restricted activity of young people (young adults and teenagers). The novelty of this contribution is derived from two specific features. One locates each geographic region in our parameter space at any stage providing for a diagnosis of the disease status in the region, and the prevailing quality of its management. The other affords a direction in which changes in strategy must be brought about for controlling the disease. Although a rational analysis for an economic reboot should be based on a considerably expanded view of the local economics, it is possible to derive some useful guidelines from our model study. To this extent, we conclude, perhaps somewhat speculatively, that our suggestion for an economic reopening may be viable if non-essential business closure is conditional, mass gathering is limited and school opening is delayed. At any rate, in the absence of such restrictive measures, the prospect of an economic recovery is less likely. For the further study for the school opening, the transmission among different age groups (i.e. teenager/adult/elderly) should be carefully considered. In addition, the effect of the policy mandate (e.g. mask wearing) on COVID-19 transmission will likely be studied.

## Methods

### Mechanics model

We consider a geometric domain *D* in which there is a total population density (number per unit area) $N_o$, containing *n* individuals (per unit area) diseased with the coronavirus. These population densities must be clearly viewed as averaged over the whole domain, which may be a county, city, state or even an entire country. One may expect steep variations of the actual number densities about their average values. Both the averages and fluctuations about them may be expected to vary with time and location, and a model that includes such dynamic effects would require a complex mathematical framework riddled with parameters not easily identified even if detailed observations can be made. Our goal here is to find a suitably simple framework to produce a mathematical model that contains a limited number of parameters which can be readily identified from gross observations. Furthermore, they should relate in some way to various strategies that may be envisaged to control the spread of infection.

The basic mechanism of spreading infection is assumed to be exposure to a viral population in the environment (exhaled droplets, aerosol, contaminated surfaces, and possibly fecal-oral contamination) contributed by all those infected with the coronavirus. Obviously, the viral population in the environment will notably vary with location but over a period of time during which individuals in motion would encounter viruses when they are in the vicinity of the infected and are thus open to catching the infection which is represented below.

3 $$\begin{aligned} {}{\text {Uninfected individual}}\xrightarrow {\text {Exposure to viral environment}}\text { Infected individual} \end{aligned}$$

The rate at which this process of infection occurs would clearly depend on the local viral population. Indeed, the viral virulence would vary significantly with location but the assumed simplicity of the model is derived from sufficient mixing of both the infected and the uninfected individuals that they see the average viral population over a period of time. The fate of infected individuals is represented by

4 $$\begin{aligned} {}{\text {Infected individual}}\xrightarrow {\text {Under treatment}}\text {Recovered or deceased individual} \end{aligned}$$

Mathematically, we represent the rate in Eq. (3) by *kV* where *V* is the viral population averaged over the domain *D*. Alternately, (1/*kV*) may be viewed as the average time for an individual to be infected. Since the transfer of infection implied in Eq. (3) occurs in multiple ways, the average time for infection must represent the mean of the average times for the different mechanisms. Thus the rate at which the infected population increases is then given by $k(N_o-n)V$, where $(N_o-n)$ represents the uninfected population density. The rate constant associated with the process of removed population is denoted as $k_r$ and $k_rn$ represents the rate at which infected people are removed by death or recovering from treatment.

We can now write a balance equation for the infected population given by

5 $$\begin{aligned} \frac{dn}{dt}=kV\left( {{N}_{o}}-n \right) -{{k}_{r}}n. \end{aligned}$$

Eq. (5) is coupled to a balance equation for $V$, the viral population which accounts for their multiplication and their random death. This is given by

6 $$\begin{aligned} \frac{dV}{dt}={{k}_{v}}n-k_v'V. \end{aligned}$$

The first term on the right-hand side accounts for viral multiplication from all diseased individuals while the second term is their first order disappearance. The model at this stage is represented by 4 parameters, $k,{{k}_{r}},{{k}_{v}},{k_v'}$. Table 2 collates their broad interpretations representing the spreading characteristics. It should be apparent that constants $k_r$ and $k_v'$ are not as sensitive to disease management as the constants *k* and $k_v$ are. For instance *k* and $k_v$ could be conceivably reduced by the use of masks and social distancing.

Insofar as the infection curve results from different processes, it is more meaningful to convert the model variables to dimensionless variables as below.

7 $$\begin{aligned} x\equiv \frac{n}{{{N}_{o}}}\text {= fraction infected},\quad y\equiv \frac{V}{{{V}_{o}}}=\text {Viral population density scaled to }{{V}_{o}},\quad \tau \equiv k{{V}_{o}}t \end{aligned}$$

As a scaled variable, $y$ is a more convenient representative of the viral population as their absolute numbers are very large. The scaled time relates absolute time to the average time it takes to infect a single person. Three dimensionless parameters emerge from the differential equations (Eqs. (1–2)) that are presented in Table 1, along with their physical interpretations.

### Peak infection rate

The peak infection rate occurs when the second derivative ${{d}^{2}}x/d{{\tau }^{2}}$vanishes along its path. It is readily shown that this occurs when

8 $$\begin{aligned} y=\frac{1}{2}\left( \frac{\gamma x}{1-x}-\beta -\gamma \right) +\frac{1}{2}\sqrt{{{\left( \frac{\gamma x}{1-x}-\beta -\gamma \right) }^{2}}+4x\left( \frac{{{\gamma }^{2}}}{1-x}+\alpha \right) }. \end{aligned}$$

The peak infection rate is obtained when the curve represented in Eq. (8) intersects the curve $\left\{ x\left( \tau \right) ,y\left( \tau \right) \right\}$. This represents the stage beyond which the infection rate will drop. It is now possible to address the much debated strategy of “flattening the curve” at a lower infected fraction. Therefore, the peak infection rate (PIR) occurs at

9 $$\begin{aligned} \text {PIR}=\frac{1}{2}\left( 1-x \right) \left\{ \left( \frac{\gamma x}{1-x}-\beta -\gamma \right) +\sqrt{{{\left( \frac{\gamma x}{1-x}-\beta -\gamma \right) }^{2}}+4x\left( \frac{{{\gamma }^{2}}}{1-x}+\alpha \right) } \right\} -\gamma x \end{aligned}$$

It will then be of interest to specify either the maximum infection rate or the fraction infected at which there is noticeable slowing of the infection rate (Figs. 2C,D of the manuscript).

### Modeling lockdown of New York city

Here, we consider the influence of lockdown policy at New York City, where the isolation is determined by the geographic constraint of five boroughs (the Bronx, Brooklyn, Manhattan, Queens, and Staten Island) within the New York City. In the strict isolation, the entire New York City D is divided into five boroughs ($D_i$, $i=1, 2,\ldots ,5$) and there being no interaction between the populations in different boroughs (Fig. 3A of the manuscript). We may model the transmission dynamics in domain $D_i$ by using Eqs. (1) and (2) with the parameters of

10 $$\begin{aligned} \alpha _i=\alpha \cdot \frac{N_{io}}{N_o},\quad \beta _i=\beta ,\quad \gamma _i=\gamma \cdot \frac{N_{io}}{N_o}, \end{aligned}$$

where ($\alpha$, $\beta$, $\gamma$) are the parameters for New York City; $N_{io}$ is the total residing population in borough *i* and $N_o$ is the total population in New York City. In Fig. 3B of the manuscript, we evaluate the lockdown strategy by comparing *x* obtained for domain *D* with $\sum _i^5x_i$.

**Table 2:** Variable definitions in model.

Notation	Variables
*n(t)*	Infected population density at any time $t$
$N_o$	Population density in the domain
$V_o$	Characteristic viral population density
*V(t)*	Viral population density at any time $t$
*k*	Rate constant for transfer of infection ${{k}^{-1}}$ is the average time for infection
$k_v$	Rate constant for production of virus from the infected $k_{v}^{-1}$is the average time for viral multiplication
$k_v'$	Rate of death of virus
$k_r$	Rate of removal of population (either by death or recovery)

## Supplementary information

Supplementary Information.

## Data Availability

The data and code that support the findings of this study are available from the corresponding author upon reasonable request. Parameters for reproducing the calculated numerical results and protocols for reproducing the numerical experiments are included in the main text and supplementary material. US coronavirus data are publically available from the New York Times GitHub source: https://raw.githubusercontent.com/nytimes/covid-19-data/master/us-counties.csv; Weather data are publicly available from NOAA Global Surface Summary of the Day (GSOD): https://data.nodc.noaa.gov/cgi-bin/iso?id=gov.noaa.ncdc:C00516; State and policy data to address coronavirus are publically available from Kaiser Family Foundation: https://www.kff.org/health-costs/issue-brief/state-data-and-policy-actions-to-address-coronavirus/.
