# Supplementary material for: A model to rate strategies for managing disease due to COVID-19 infection
Source: Sci Rep. 2020 Dec 31;10:22435. doi: 10.1038/s41598-020-79817-7 (PMC7775474; doi:10.1038/s41598-020-79817-7)
Supplement: Supplementary file 1 — Supplementary Information. [file 41598_2020_79817_MOESM1_ESM.pdf]

Supplementary Material

Title
A Model to Rate Strategies for Managing Disease due to COVID-19 Infection

Authors
Shiyan Wang[1] and Doraiswami Ramkrishna[1]*

Affiliations
[1]Davidson School of Chemical Engineering, Purdue University, West Lafayette Indiana, 47907, USA
*To whom correspondence should be addressed; E-mail: ramkrish@purdue.edu.

This file includes:

Materials and Methods
Supplementary Text
Figs. S1 to S4
Tables S1 to S3
References and Notes

Materials and Methods

Data. Daily coronavirus infection data (county by county) in United States came from New York Times GitHub source (*21*). We extracted daily positive infection numbers from 21st January 2020 to 25th April 2020. To estimate the effect of weather (county by county) on the transmissibility of the coronavirus, we used the weather data for 2020 from NOAA Global Surface Summary of the Day (GSOD) (*22*). To study the state orders and policies for addressing the coronavirus (state by state in US), we extracted data from Kaiser Family Foundation (*23*).

Discussion of R0. In the Fig.1(A) of the manuscript, the P1 duration reveals the period of pathogen transmission with limited prevention in the United States. The early state of virus transmissibility can be characterized by `R-naught' (R0), which is the basic reproduction number for the expected number of cases directly generated by one case in a population where all individuals are susceptible to infection, $n(t) = (0)\cdot \mathrm{R0}^{t/s}$; s is the serial interval (for COVID-19, its median $\bar{s} \approx 3.96$ days (*8*)). Our model estimates $\mathrm{R0} \sim e^{\bar{s}/T_{\inf}}$ where $\lambda = \left[-(\beta+\gamma)+\sqrt{(\beta-\gamma)^2+4\alpha}\right]/2$ is the largest eigenvalue using the approach of next generation matrix. Therefore, our estimate of R0 is about 2.8 (the median from data is 2.75; our model is 2.90) whose transmission is stronger than influenza (R0:1.4-1.6) (*9*) and weaker than Measles (R0:12-18) (*10*).

Correlation of Weather vs. $\tilde{x}$ and α/β. Fig. S1(A) shown below indicates that the correlation between $\tilde{x}$ and mean temperature (mean) is insignificant (p=0.56>0.05). Figs. S1(B) shows the relationship between $\tilde{x}$ and mean dew point (humidity). For the virulence (α/β), both temperature and dew point is lack of significance in relevance (Figs. S1(C, D).

During the machine learning, we aim to include enough significant independent variables. We do not incorporate both temperature and dew point in our regression model because of the insignificant correlation. On the other hand, based on our analysis on the population and population density in Figs. 2(A, B) of the manuscript, which shows strong correlation to α/β and $\tilde{x}$, respectively. We have incorporate both population and population density in the machine learning model.

Machine Learning Model: Evaluation of Policy. In this section, we elaborate our procedure of applying regression model to 2 important indicators $\tilde{x}$ (projected total infection fraction) and α/β (virulence) using machine learning. This section includes: (I) data definition, (II) coding system for

categorical values, (III) Feature reduction, (IV) regression and cross validation, (V) prediction of policy change following economy opening on the coronavirus transmission.

(I) Data Definition: Since March 16th when the US administration declares the '15 days to slow the spread' policy, local government in each state have declared more than 300 executive orders, where the general policy measures can be categorized into 7 aspects:

1) Stay at home order:
    a. Statewide
    b. High-risk groups
    c. Rolled back to high-risk groups
    d. Lifted
    e. N/A
2) Easing social distancing measure:
    a. No
    b. Yes
3) Mandatory quarantine for travelers:
    a. All travelers
    b. All air travelers
    c. From certain states
    d. Rolled back to certain states
    e. Other
    f. Lifted
    g. N/A
4) Non-essential Business closure:
    a. All non-essential business closed
    b. Some non-essential business closed
    c. Some non-essential businesses permitted to reopen with reduced capacity
    d. Some non-essential businesses permitted to reopen
    e. All non-essential businesses permitted to reopen with reduced capacity
    f. N/A
5) Gathering ban:
    a. All gathers prohibited
    b. Expanded to 25+ people prohibited
    c. Expanded to 20+ people prohibited

d. 10+ people prohibited
e. Expanded to 10+ people prohibited
f. Other
g. Lifted
h. N/A

6) School closure:
   a. Closed for school year
   b. Recommended closure for school year
   c. Closed
   d. N/A
7) Restaurant limits:
   a. Closed except for takeout/delivery
   b. Limited dine-in service
   c. Reopened to dine-in service with capacity limits
   d. Reopened to dine-in service
   e. N/A

Note that 'N/A' represents that no action is taken. For machine learning training, our data of policies are collected on May 5th for include 50 states and Washington D.C. (see data sample in Table S3-1)

<u>(II) Coding System for Categorical Variables in Regression Analysis</u>: For each policy as a categorical variable, we using *simple coding approach* to convert the categorical variable into a series of variables for regression analysis. In simple coding method, we define a reference level for each categorical variable. For instance, in variable 'Stay at home order', we treat the item 'N/A' as the reference level. Therefore, using simple coding system, the parameters can be defined in Table S1. In the Table S1, the level of the categorical variable that is coded as '-1/5' in all of new variables is the reference level. Instead of categorical variable, we enter new variables into regression. We should note that during the regression, the weight of variable 'x1' would be the weight for 'Statewide' minus the weight for 'N/A'. Similarly, other categorical variables can be transformed to new variables. For every categorical variable, we always choose the last option as the reference level.

(III) Feature Selection: P-value & VIF: In linear regression, the predictor is the projected total infection percentage ($\tilde{x}$). We perform the linear regression (ordinary least squares regression) against the individual categorical variable and calculate P-value and VIF (Variance inflation factor). P-value represent the null-hypothesis and we require $p<0.05$ to be significant. On the other hand, VIF quantifies the severity of the collinearity during regression analysis (VIF<5). For each categorical variable, we list their performance in Table S2. Based on above analysis, for regression of $\tilde{x}$, variables (i), (ii), (iii), (vii) are insignificant correlation. Variables (iv), (v) and (vi) are significant. Similarly, we can perform regression of individual variables against α/β.

(IV) Linear Regression & Cross Validation: Next, we include all significant variables in the linear regression model, where the variables are associated with categorical variables (iv), (v) and (vi). Total number of variables associated with categorical variables are 15. By performing the regression with all fifteen variables, we found that for categorical variable (iv), the only significant variable is ‘x1’. By excluding all other variables with categorical variable (iv), the overall performance of the model remains unchanged with total 11 variables. Additionally, we include another significant continuous variable ‘population density’. With a total of 12 variables, we performed cross validation (10-fold) to examine the significance of our model. In general, all 12 variables are significant in determining $\tilde{x}$ (see Fig. S2(B) for p-value). By examine the weight of each variable, we know that all features are important to determine $\tilde{x}$. The model has a R-square value around 0.7, indicating 70% accuracy in prediction. On the other hand, for α/β, we only include the population and variables (iv) (see Fig. S3).

(V) Prediction of Policy Change Following Economy Opening on Coronavirus Transmission: Following policy of “Opening Up America Again” by the white house administration, states have revised their policy on social distancing. We collect policy data on June 5, 2020 when all states (50 + Washington D.C.; see data sample in Table S3-2) have eased the measures of social distancing. Using the trained machine learning model, we predict both α/β and $\tilde{x}$ based on the changes of policies. We identify ‘emerging risk of state’ based on the elevation of either α/β (states marked as red filled circle in Fig. 4(C) of the manuscript) or $\tilde{x}$ (states marked as green filled star in Fig. 4(C) of the manuscript).

Supplementary Text

<u>Mechanics Model Implementation.</u> We solve the Eqs. (1-2) of the manucript using ode23tb subroutine from Matlab because of the stiffness of the nonlinear equations. To fit parameters α / β , $kV_o = 1/T_{\inf}$, and γ . We consider data from leading county of fifty states and Washington D.C. Next, we defined an objective function and minimized it using fminsearch subroutine.

To consider the uncertainty of the parameter fitting, we perform Monte Carlo simulations (sample size is 50) to improve the confidence of our fitting. We should note that: in Fig. 1(C), the plot of the model used the median of all realization; In Figs. 2(A, B), all parameters used in the model are median of the distributions; In Figs. 3(B), the plot of the model used the median of all realization; In Fig. 3(C), the solid line is the median of the distribution and the upper and lower limits of the shaded area are the maximum and minimum of the distribution.

In our mechanistic model, we consider the both infection dynamics of infected population and virulence environment, where the similar mechanism has been applied to study Malaria (i.e., Malaria Model (*24*,*25*)). On the other hand, the transmissibility dynamics estimated by the SIR model considers the factor of infected population. Secondly, our model aims to minimize the fitting parameters against the infection curve; other compartmental models in epidemiology (e.g., SEIR), they could result in many parameters fitting, which may lead to the overfitting of the data (*26*).

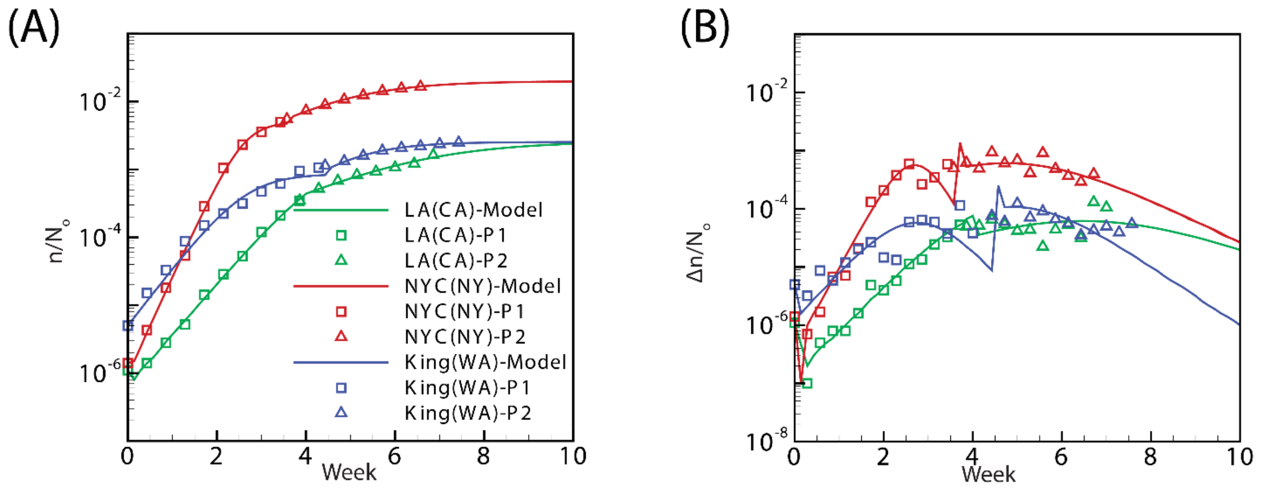

Fig. S1(**A** and **B**) **Fitting of mechanistic model to the coronavirus infection data.** The dynamic evolution of (B) infected population density n and (C) daily increment Δn are scaled by the county population density N_o; the data are associated with three counties (e.g., Los Angeles county in California State, New York City in New York State, and King county in Washington State); The zeroth week is set at the moment when the total number of infections in individual counties is ten.

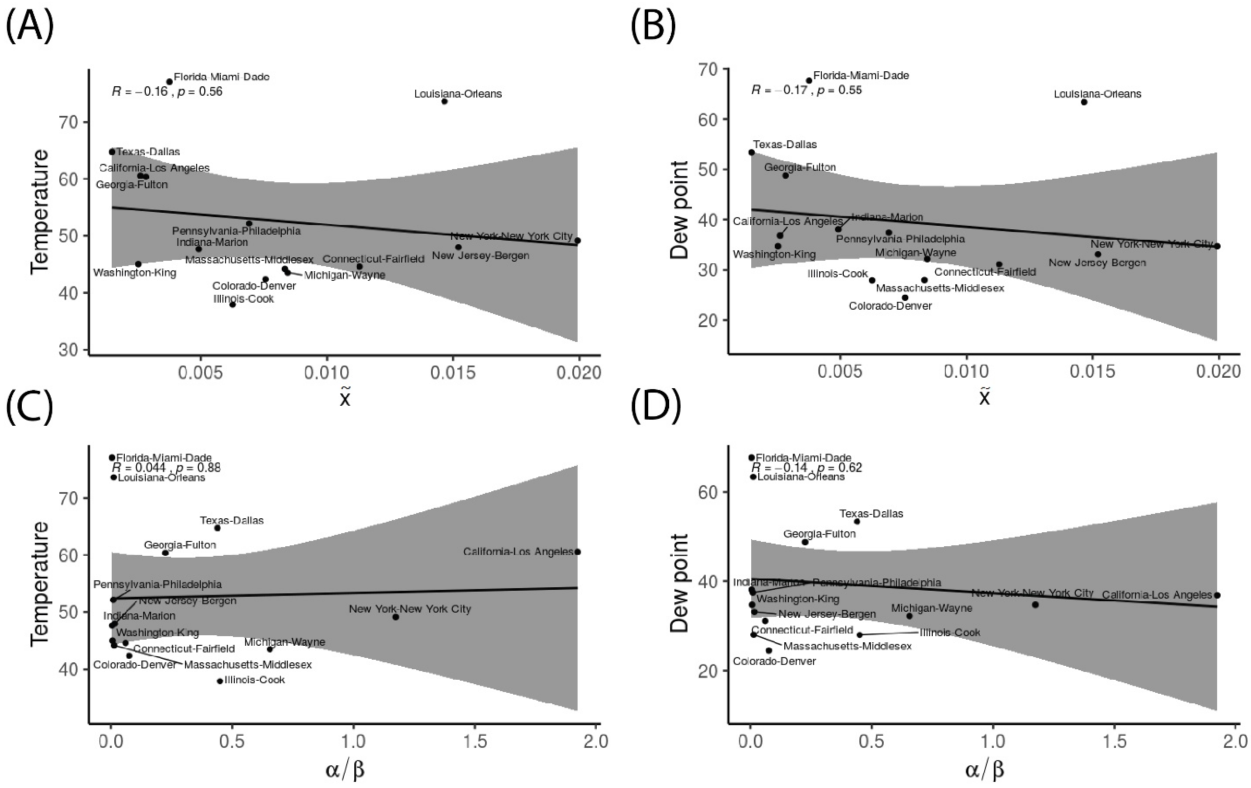

Fig. S2. Impact of weather on the model parameters. (A **and** B) **The correlation between (A) mean temperature vs. the projected infection fraction (** $\tilde{x}$ **), and (B) dew point vs. the projected infection fraction (** $\tilde{x}$ **).** (C **and** D) **The correlation between (C) mean temperature vs. the virulence parameter (** α / β **), and (B) dew point vs. the virulence parameter (** α / β **). The sample size is 15.**

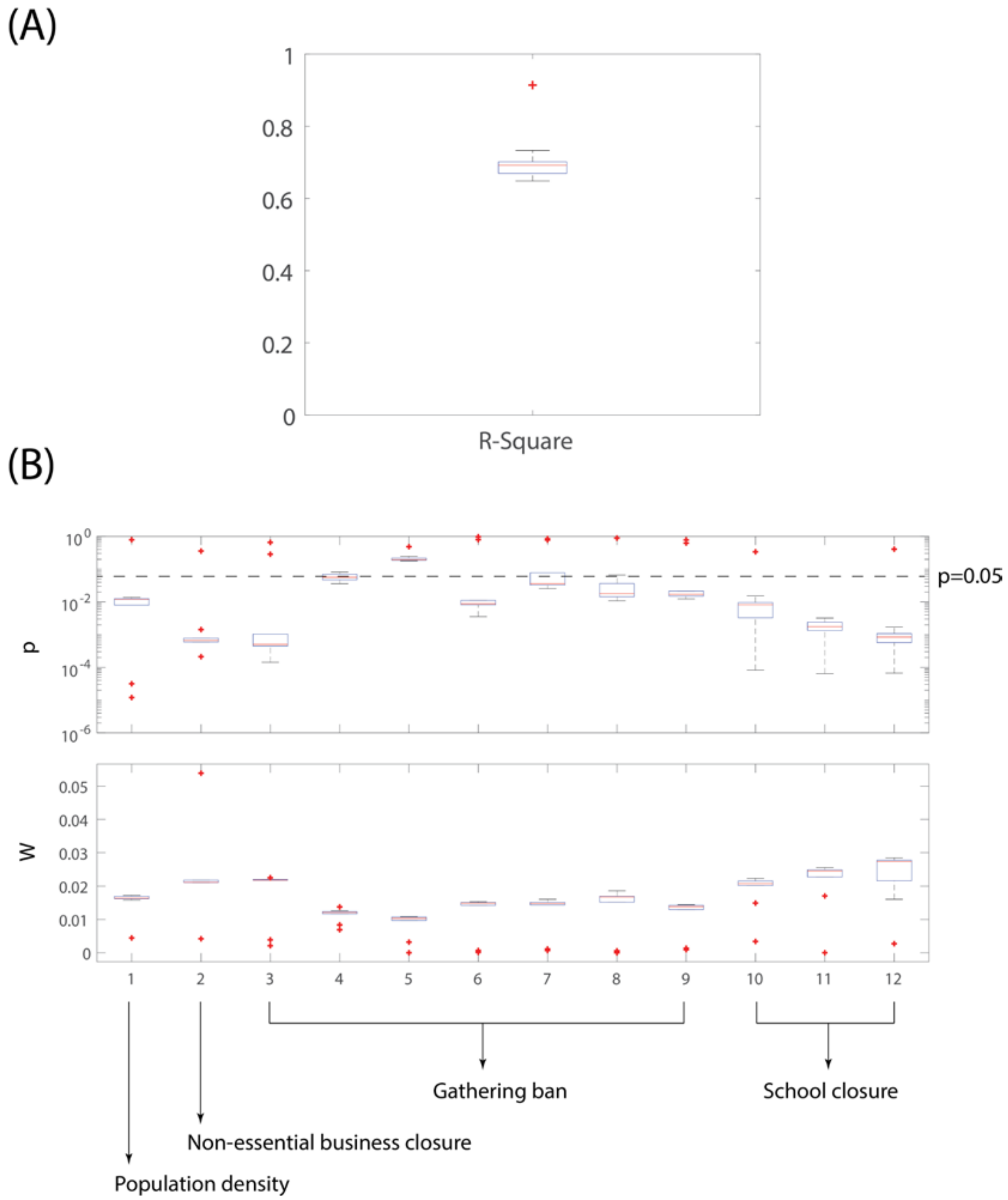

Fig. S3. Performance of regression of $\tilde{x}$ using cross validation. (A) **The boxplot of R-Square using cross validation.** (B) **The boxplot for p-value and weight of 12 variables using cross validation.**

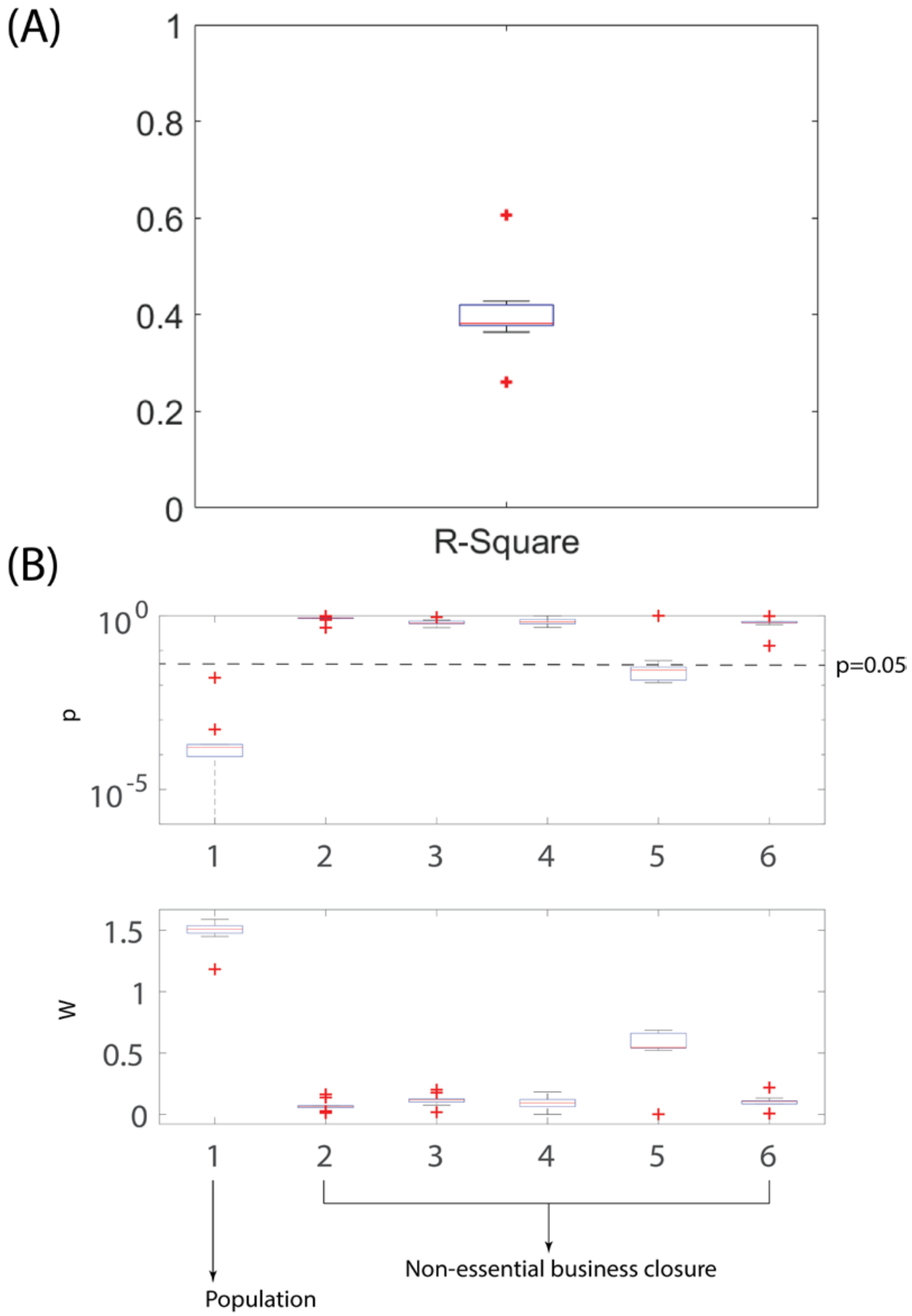

Fig. S4. Performance of regression of α/β using cross validation. (A) **The boxplot of R-Square using cross validation.** (B) **The boxplot for p-value and weight of 12 variables using cross validation.**

Table S1. Coding System for Categorical Variable ‘Stay at home order’

**Feature ‘Stay at home order’**	**New Variable (x1)**	**New Variable (x2)**	**New Variable (x3)**	**New Variable (x4)**
‘Statewide’	4/5	-1/5	-1/5	-1/5
‘High-risk groups’	-1/5	4/5	-1/5	-1/5
‘Rolled back to high-risk groups’	-1/5	-1/5	4/5	-1/5
‘Lifted’	-1/5	-1/5	-1/5	4/5
‘N/A’	-1/5	-1/5	-1/5	-1/5

Table S2-1. P-values and VIF coefficients for $\tilde{x}$ against all categorical values

(i) 'Stay at home order'

Variables	P-value	VIF
**x1**	0.0438	2.41
**x2**	0.3069	1.28
**x3**	0.2336	1.14
**x4**	0.0818	2.55
**P-value for regression model: 0.335**		

(ii) 'Easing social distancing measure'

Variables	P-value	VIF
**x1**	0.879	1
**P-value for regression model: 0.879**		

(iii) 'Mandatory quarantine for travelers'

Variables	P-value	VIF
**x1**	0.6360	1.0154
**x2**	0.7244	1.0154
**x3**	0.7277	1.0154
**x4**	0.3957	1.0714
**x5**	0.6351	1.0154
**x6**	0.2189	1.0910
**P-value for regression model: 0.695**		

Table S2-2. P-values and VIF coefficients for $\tilde{x}$ against all categorical values

(iv) ‘Non-essential Business closure’

Variables	P-value	VIF
**x1**	8.7586e-05	1.2810
**x2**	0.62019	1.9673
**x3**	0.85621	2.2941
**x4**	0.2553	1.7647
**x5**	0.66362	2.5556
**P-value for regression model: 0.00131**		

(i) ‘Gathering ban’

Variables	P-value	VIF
**x1**	0.0019359	2.7647
**x2**	0.0043698	2.3529
**x3**	0.017636	1.4706
**x4**	1.3066e-05	6.8824
**x5**	0.00038911	1.9216
**x6**	0.00073714	1.9216
**x7**	8.2682e-05	5.5882
**P-value for regression model: 0.00253**		

(ii) ‘School closure’

Variables	P-value	VIF
**x1**	0.24445	8.8431
**x2**	0.05413	6.1765
**x3**	0.041329	11.6667
**P-value for regression model: 0.0261**		

Table S2-3. P-values and VIF coefficients for $\tilde{x}$ against all categorical values

(iii) 'Restaurant limits'

Variables	P-value	VIF
**x1**	0.55178	1.9608
**x2**	0.67832	10.8824
**x3**	0.4261	2.8824
**x4**	0.65758	12.0000
**P-value for regression model: 0.92**		

Table S3-1. Data sample (n=6) for policies are collected on May 5th (leading county of 50 states and Washington D.C.)

No	state	county	Stay at home order	Social distancing measure	Mandatory quara travelers	Non-essen bus closure	Gathering ban	School closure	Restaurant limits
1	New York	New York City	Statewide	NO	NA	All Non-Essential Businesses Closed	All Gatherings Prohibited	Closed for School Year	Closed Except for Takeout/Delivery
2	New Jersey	Bergen	Statewide	NO	NA	Some Non-Essential Businesses Closed	All Gatherings Prohibited	Closed	Closed Except for Takeout/Delivery
3	Michigan	Wayne	Statewide	YES	NA	Some Non-Essential Businesses Permitted to Reopen	All Gatherings Prohibited	Closed for School Year	Closed Except for Takeout/Delivery
4	California	Los Angeles	Statewide	NO	NA	All Non-Essential Businesses Closed	All Gatherings Prohibited	Recommended Closure for School Year	Closed Except for Takeout/Delivery
5	Louisiana	Orleans	Statewide	NO	NA	Some Non-Essential Businesses Closed	10+ People Prohibited	Closed for School Year	Closed Except for Takeout/Delivery
6	Massachusetts	Middlesex	Statewide	NO	All Travelers	All Non-Essential Businesses Closed	10+ People Prohibited	Closed for School Year	Closed Except for Takeout/Delivery

Table S3-2. Data sample (n=6) for policies are collected on June 5th (leading county of 50 states and Washington D.C.)

No	state	county	Stay at home order	Social distancing measure	Mandatory quara travelers	Non-essen bus closure	Gathering ban	School closure	Restaurant limits
1	New York	New York City	Statewide	YES	NA	Some Non-Essential Businesses Permitted to Reopen with Reduced Capacity	Expanded to 20+ People Prohibited	Closed for School Year	Closed Except for Takeout/Delivery
2	New Jersey	Bergen	Statewide	YES	NA	Some Non-Essential Businesses Permitted to Reopen with Reduced Capacity	Expanded to 20+ People Prohibited	Closed	Closed Except for Takeout/Delivery
3	Michigan	Wayne	Lifted	YES	NA	Some Non-Essential Businesses Permitted to Reopen	Expanded to 25+ People Prohibited	Closed for School Year	Closed Except for Takeout/Delivery
4	California	Los Angeles	Statewide	YES	NA	Some Non-Essential Businesses Permitted to Reopen with Reduced Capacity	All Gatherings Prohibited	Recommended Closure for School Year	Closed Except for Takeout/Delivery
5	Louisiana	Orleans	Lifted	YES	NA	Some Non-Essential Businesses Permitted to Reopen with Reduced Capacity	Lifted	Closed for School Year	Reopened to Dine-in Service with Capacity Limits
6	Massachusetts	Middlesex	Rolled Back to High Risk Groups	YES	All Travelers	Some Non-Essential Businesses Permitted to Reopen	10+ People Prohibited	Closed for School Year	Closed Except for Takeout/Delivery

References and Notes

1. Chinazzi, M. et al. The effect of travel restrictions on the spread of the 2019 novel coronavirus (covid-19) outbreak. Science 368, 395–400 (2020).
2. Ferretti, L. et al. Quantifying sars-cov-2 transmission suggests epidemic control with digital contact tracing. Science 368 (2020).
3. Bourouiba, L. Turbulent gas clouds and respiratory pathogen emissions: potential implications for reducing transmission of covid-19. Jama 323, 1837–1838 (2020).
4. Guo, Z.-D. et al. Aerosol and surface distribution of severe acute respiratory syndrome coronavirus 2 in hospital wards, wuhan, china, 2020. Emerg Infect Dis 26, 10–3201 (2020).
5. Lauer, S. A. et al. The incubation period of coronavirus disease 2019 (covid-19) from publicly reported confirmed cases: estimation and application. Annals internal medicine 172, 577–582 (2020).
6. Lipsitch, M. et al. Transmission dynamics and control of severe acute respiratory syndrome. Science 300, 1966–1970 (2003).
7. Repici, A. et al. Coronavirus (covid-19) outbreak: what the department of endoscopy should know. Gastrointest. Endoscopy (2020).
8. Lan, L. et al. Positive rt-pcr test results in patients recovered from covid-19. Jama 323, 1502–1503 (2020).
9. Fraser, C. et al. Pandemic potential of a strain of influenza a (h1n1): early findings. science 324, 1557–1561 (2009).
10. Guerra, F. M. et al. The basic reproduction number (r0) of measles: a systematic review. The Lancet Infect. Dis. 17, e420–e428 (2017).
11. Grein, J. et al. Compassionate use of remdesivir for patients with severe covid-19. New Engl. J. Medicine (2020).
12. Baker, R. E., Yang, W., Vecchi, G. A., Metcalf, C. J. E. & Grenfell, B. T. Susceptible supply limits the role of climate in the early sars-cov-2 pandemic. Science (2020).
13. Eichenbaum, M. S., Rebelo, S. & Trabandt, M. The macroeconomics of epidemics. Tech. Rep., National Bureau of Economic Research (2020).
14. Tian, S. et al. Characteristics of covid-19 infection in beijing. J. Infect. (2020).
15. Day, M. Covid-19: identifying and isolating asymptomatic people helped eliminate virus in italian village. BMJ: Br. Med. J. (Online) 368 (2020).
16. World Health Organization and others Environmental cleaning and disinfection in non-health-care settings in the context of COVID-19. Manila: WHO Reg. Off. for West. Pac. (2020).
17. World Health Organization and others Infection prevention during transfer and transport of patients with suspected COVID-19 requiring hospital care. Manila: WHO Reg. Off. for West. Pac. (2020).
18. Siddiqui, Mohammad Khubeb and Morales-Menendez, Ruben and Gupta, Pradeep Kumar and Iqbal, HM and Hussain, Fida and Khatoon, Khudeja and Ahmad, Sultan Correlation between temperature and COVID-19 (suspected, confirmed and death) cases based on machine learning analysis. J Pure Appl Microbiol 14 (2020).
19. Singh, S. and Roy, M. D. and Sinha, K. and Parveen, S. and Sharma, G. and Joshi, G. Impact of COVID-19 and lockdown on mental health of children and adolescents: A narrative review with recommendations. Psychiatry Res. 113429 (2020).
20. Son, C. and Hegde, S. and Smith, A. and Wang, X. and Sasangohar, F. Effects of COVID-19 on college students' mental health in the United States: Interview survey study. J. Med. Internet Res. 22 (2020).
21. New York Times github source, *US coronavirus data* (2020); https://raw.githubusercontent.com/nytimes/covid-19-data/master/us-counties.csv.
22. NOAA Global Surface Summary of the Day, *Weather data for 2020* (2020); https://data.nodc.noaa.gov/cgi-bin/iso?id=gov.noaa.ncdc:C00516.
23. Kaiser Family Foundation, *State Data and Policy Actions to Address Coronavirus* (2020); https://www.kff.org/health-costs/issue-brief/state-data-and-policy-actions-to-address-coronavirus/.
24. J. H. Jones, Notes on R0. *California: Department of Anthropological Sciences* **323** (2007).
25. D. L. Smith, F. E. McKenzie, R. W. Snow, S. I. Hay, Revisiting the basic reproductive number for malaria and its implications for malaria control. *PLoS biology* **5**(3) (2007).
26. M. Pollicott, H. Wang, H. Weiss, Extracting the time-dependent transmission rate from infection data via solution of an inverse ODE problem. *J. Boil. Dynam.* **6**(2), 509-523 (2012).
